# Supplementary material for: Pyruvate Dehydrogenase Kinase 1 inhibition mediated oxidative phosphorylation enhancement in cartilage promotes osteoarthritis progression
Source: BMC Musculoskelet Disord. 2023 Jul 20;24:597. doi: 10.1186/s12891-023-06585-6 (PMC10357736; doi:10.1186/s12891-023-06585-6)
Supplement: Supplementary file 1 — Additional file 1. [file 12891_2023_6585_MOESM1_ESM.zip › KEGG.Gsea.1653622432464/KEGG_AMINO_SUGAR_AND_NUCLEOTIDE_SUGAR_METABOLISM.html]

Details for gene set KEGG\_AMINO\_SUGAR\_AND\_NUCLEOTIDE\_SUGAR\_METABOLISM[GSEA]

|  || Dataset | 55457\_collapsed\_to\_symbols.cls.cls #OA\_versus\_normal.cls.cls #OA\_versus\_normal\_repos |
| Phenotype | cls.cls#OA\_versus\_normal\_repos |
| Upregulated in class | OA |
| GeneSet | KEGG\_AMINO\_SUGAR\_AND\_NUCLEOTIDE\_SUGAR\_METABOLISM |
| Enrichment Score (ES) | 0.44143814 |
| Normalized Enrichment Score (NES) | 1.6824048 |
| Nominal p-value | 0.007518797 |
| FDR q-value | 0.027037522 |
| FWER p-Value | 0.333 |
Table: GSEA Results Summary

  

Fig 1: Enrichment plot: KEGG\_AMINO\_SUGAR\_AND\_NUCLEOTIDE\_SUGAR\_METABOLISM      
 Profile of the Running ES Score & Positions of GeneSet Members on the Rank Ordered List

  

| SYMBOL | TITLE | RANK IN GENE LIST | RANK METRIC SCORE | RUNNING ES | CORE ENRICHMENT || 1 | CYB5R1 | cytochrome b5 reductase 1 [Source:HGNC Symbol;Acc:HGNC:13397] | 30 | 1.052 | 0.0809 | Yes |
| 2 | UXS1 | UDP-glucuronate decarboxylase 1 [Source:HGNC Symbol;Acc:HGNC:17729] | 91 | 0.926 | 0.1496 | Yes |
| 3 | GPI | glucose-6-phosphate isomerase [Source:HGNC Symbol;Acc:HGNC:4458] | 166 | 0.842 | 0.2105 | Yes |
| 4 | CMAS | cytidine monophosphate N-acetylneuraminic acid synthetase [Source:HGNC Symbol;Acc:HGNC:18290] | 198 | 0.822 | 0.2732 | Yes |
| 5 | MPI | mannose phosphate isomerase [Source:HGNC Symbol;Acc:HGNC:7216] | 255 | 0.781 | 0.3306 | Yes |
| 6 | NAGK | N-acetylglucosamine kinase [Source:HGNC Symbol;Acc:HGNC:17174] | 256 | 0.780 | 0.3924 | Yes |
| 7 | NPL | N-acetylneuraminate pyruvate lyase [Source:HGNC Symbol;Acc:HGNC:16781] | 1155 | 0.485 | 0.3610 | Yes |
| 8 | HEXB | hexosaminidase subunit beta [Source:HGNC Symbol;Acc:HGNC:4879] | 1434 | 0.438 | 0.3740 | Yes |
| 9 | AMDHD2 | amidohydrolase domain containing 2 [Source:HGNC Symbol;Acc:HGNC:24262] | 1595 | 0.415 | 0.3944 | Yes |
| 10 | GALT | galactose-1-phosphate uridylyltransferase [Source:HGNC Symbol;Acc:HGNC:4135] | 1666 | 0.404 | 0.4209 | Yes |
| 11 | GNPDA1 | glucosamine-6-phosphate deaminase 1 [Source:HGNC Symbol;Acc:HGNC:4417] | 1795 | 0.385 | 0.4414 | Yes |
| 12 | HEXA | hexosaminidase subunit alpha [Source:HGNC Symbol;Acc:HGNC:4878] | 3025 | 0.247 | 0.3655 | No |
| 13 | FPGT | fucose-1-phosphate guanylyltransferase [Source:HGNC Symbol;Acc:HGNC:3825] | 3259 | 0.225 | 0.3652 | No |
| 14 | HK3 | hexokinase 3 [Source:HGNC Symbol;Acc:HGNC:4925] | 3792 | 0.178 | 0.3379 | No |
| 15 | PGM1 | phosphoglucomutase 1 [Source:HGNC Symbol;Acc:HGNC:8905] | 3990 | 0.157 | 0.3350 | No |
| 16 | GMDS | "GDP-mannose 4,6-dehydratase [Source:HGNC Symbol;Acc:HGNC:4369]" | 4151 | 0.142 | 0.3338 | No |
| 17 | PMM1 | phosphomannomutase 1 [Source:HGNC Symbol;Acc:HGNC:9114] | 4238 | 0.134 | 0.3377 | No |
| 18 | GALK2 | galactokinase 2 [Source:HGNC Symbol;Acc:HGNC:4119] | 4945 | 0.076 | 0.2889 | No |
| 19 | CYB5R3 | cytochrome b5 reductase 3 [Source:HGNC Symbol;Acc:HGNC:2873] | 4986 | 0.074 | 0.2916 | No |
| 20 | PGM3 | phosphoglucomutase 3 [Source:HGNC Symbol;Acc:HGNC:8907] | 5290 | 0.049 | 0.2719 | No |
| 21 | RENBP | renin binding protein [Source:HGNC Symbol;Acc:HGNC:9959] | 5653 | 0.019 | 0.2453 | No |
| 22 | GALK1 | galactokinase 1 [Source:HGNC Symbol;Acc:HGNC:4118] | 5785 | 0.009 | 0.2358 | No |
| 23 | GFPT1 | glutamine--fructose-6-phosphate transaminase 1 [Source:HGNC Symbol;Acc:HGNC:4241] | 5847 | 0.005 | 0.2315 | No |
| 24 | GFPT2 | glutamine-fructose-6-phosphate transaminase 2 [Source:HGNC Symbol;Acc:HGNC:4242] | 5849 | 0.005 | 0.2318 | No |
| 25 | HK1 | hexokinase 1 [Source:HGNC Symbol;Acc:HGNC:4922] | 6728 | -0.058 | 0.1681 | No |
| 26 | GMPPA | GDP-mannose pyrophosphorylase A [Source:HGNC Symbol;Acc:HGNC:22923] | 7039 | -0.083 | 0.1506 | No |
| 27 | GFUS | GDP-L-fucose synthase [Source:HGNC Symbol;Acc:HGNC:12390] | 7317 | -0.103 | 0.1372 | No |
| 28 | NANS | N-acetylneuraminate synthase [Source:HGNC Symbol;Acc:HGNC:19237] | 7530 | -0.121 | 0.1303 | No |
| 29 | CHIT1 | chitinase 1 [Source:HGNC Symbol;Acc:HGNC:1936] | 8207 | -0.172 | 0.0913 | No |
| 30 | UGP2 | UDP-glucose pyrophosphorylase 2 [Source:HGNC Symbol;Acc:HGNC:12527] | 8735 | -0.211 | 0.0671 | No |
| 31 | PMM2 | phosphomannomutase 2 [Source:HGNC Symbol;Acc:HGNC:9115] | 9408 | -0.265 | 0.0358 | No |
| 32 | CHIA | chitinase acidic [Source:HGNC Symbol;Acc:HGNC:17432] | 9641 | -0.287 | 0.0405 | No |
| 33 | HK2 | hexokinase 2 [Source:HGNC Symbol;Acc:HGNC:4923] | 10030 | -0.325 | 0.0360 | No |
| 34 | GCK | glucokinase [Source:HGNC Symbol;Acc:HGNC:4195] | 10137 | -0.335 | 0.0543 | No |
| 35 | UGDH | UDP-glucose 6-dehydrogenase [Source:HGNC Symbol;Acc:HGNC:12525] | 10279 | -0.349 | 0.0709 | No |
| 36 | UAP1 | UDP-N-acetylglucosamine pyrophosphorylase 1 [Source:HGNC Symbol;Acc:HGNC:12457] | 11131 | -0.447 | 0.0402 | No |
| 37 | GALE | UDP-galactose-4-epimerase [Source:HGNC Symbol;Acc:HGNC:4116] | 11227 | -0.461 | 0.0692 | No |
| 38 | GNE | glucosamine (UDP-N-acetyl)-2-epimerase/N-acetylmannosamine kinase [Source:HGNC Symbol;Acc:HGNC:23657] | 12538 | -0.767 | 0.0281 | No |
Table: GSEA details [plain text format]

  

Fig 2: KEGG\_AMINO\_SUGAR\_AND\_NUCLEOTIDE\_SUGAR\_METABOLISM      
 Blue-Pink O' Gram in the Space of the Analyzed GeneSet

  

Fig 3: KEGG\_AMINO\_SUGAR\_AND\_NUCLEOTIDE\_SUGAR\_METABOLISM: Random ES distribution      
 Gene set null distribution of ES for **KEGG\_AMINO\_SUGAR\_AND\_NUCLEOTIDE\_SUGAR\_METABOLISM**
